# *Chlorella vulgaris* as a Nutraceutical Source for Broilers: Improving Meat Quality and Storage Oxidative Status

**DOI:** 10.3390/foods13152373

**Published:** 2024-07-27

**Authors:** Iulia Varzaru, Arabela Elena Untea, Tatiana Dumitra Panaite, Raluca Turcu, Mihaela Saracila, Petru Alexandru Vlaicu, Alexandra Gabriela Oancea

**Affiliations:** 1 Feed and Food Quality Department, National Research and Development Institute for Biology and Animal Nutrition, Calea Bucuresti, No.1, 077015 Balotesti, Romania; arabela.untea@ibna.ro (A.E.U.); raluca.turcu@ibna.ro (R.T.); mihaela.saracila@ibna.ro (M.S.); alexandru.vlaicu@outlook.com (P.A.V.); alexandra.oancea@ibna.ro (A.G.O.); 2 Department of Nutrition Physiology, National Research and Development Institute for Biology and Animal Nutrition, Calea Bucuresti, No.1, 077015 Balotesti, Romania; tatiana.panaite@ibna.ro

**Keywords:** microalgae, bioactive nutrients, meat quality, antioxidants, PUFA, oxidative stability

## Abstract

This study aimed to assess the impact of *Chlorella vulgaris* supplementation in broilers’ diet, alone or in combination with vitamin E, on meat quality parameters, nutritional value, and oxidative stability during storage time. An experiment was conducted on 180 COBB 500 broiler chickens (14 days old), assigned into six treatments, following a 2 × 3 factorial arrangement. A corn–soybean meal diet was supplemented with three levels of *C. vulgaris* (0% in group C1, 1% in E1, 2% in E2), two levels of vitamin E (0% in C1, 250 ppm in C2), and a combination of them (1% *C. vulgaris* + 250 ppm vitamin (E3), 2% *C. vulgaris* + 250 ppm vitamin (E4)). Dietary incorporation of *C. vulgaris*, including those supplemented with vitamin E, resulted in a significant increase in meat protein content. DPA and DHA levels increased by 2.01-fold and 1.60-fold in the 2% *C. vulgaris* + vitamin E group. The PUFA/SFA ratio was increased across all dietary treatments (*p* < 0.0001). HPI and h/H registered the highest values as a result of 2% *C. vulgaris* supplementation, being linked with a positive effect in lowering cholesterol levels. Supplementation with 2% *C. vulgaris* and vitamin E exhibited a 1.45-fold increase in vitamin E concentration in thigh meat compared to the control group, being the highest level registered in thigh meat in this experiment. Metmyoglobin concentrations registered lower values in the thigh meat of the experimental groups, while deoxymyoglobin increased in the same groups when compared to the control group. The inclusion of *C. vulgaris* (1% and 2%) in combination with vitamin E (250 mg/kg) in broiler diets exhibited the best prevention of lipid oxidation after 7 days of refrigerated storage, defined by the highest efficiency factors assessed in terms of secondary oxidation products.

## 1. Introduction

Microalgae are a diverse group of photosynthetic organisms that are known as a source of valuable bioactive compounds with numerous benefits for applications in the pharmaceuticals, food, cosmetics, and bioenergy sectors [1]. *Chlorella vulgaris* is a unicellular green microalga in the division Chlorophyta and a rich source of high-quality protein due to the important concentrations of essential amino acids. The protein content can range between 42 and 65.5% [2,3], with a lysine level of up to 13.2% [4]. *C. vulgaris* also contains important amounts of lipids, with a high content of polyunsaturated fatty acids and carbohydrates, minerals, vitamins, phenolic compounds, and pigments, such as chlorophylls and carotenoids [5,6]. During the final stage of their metabolism, microalgae undergo a transition to the carotenoid production stage, accumulating elevated quantities of pigments [7]. Carotenoids frequently present in *C. vulgaris* biomass include β-carotene, a precursor of vitamin A, along with lutein, astaxanthin, canthaxanthin, and violaxanthin. Chlorophylls are the predominant pigments within *C. vulgaris* cells, surpassing synthetic dyes and being a preferred alternative for food and cosmetic applications [8]. Additionally, chlorophyll exhibits therapeutic properties that are beneficial for ulcer treatment and liver recovery, promoting enhanced cell growth and repair [9]. Carotenoids and chlorophylls possess notable antioxidant and radical scavenging activity and have transitioned from being regarded solely as bioactive compounds to emerging as biomarkers signaling the onset of diseases associated with oxidative stress [10]. Moreover, *C. vulgaris* cells contain phenolic compounds recognized for their antioxidant, antifungal, and antibacterial activity. Additionally, it has been reported that the presence of phenolics in *C. vulgaris* formulations contributes to their antidiabetic effects [11].

Microalgae cultivation is recognized as an environmentally friendly process [12] because it requires simple conditions (CO_2_ and sunlight) and can be carried out in non-agricultural lands. Furthermore, microalgae cultivation does not necessitate the use of pesticides, unlike conventional crops, and it yields higher production outputs [13]. As photosynthetic organisms, microalgae efficiently convert atmospheric carbon dioxide into high-value compounds, playing a role in reducing CO_2_ levels in the atmosphere [14].

Due to its rich composition of essential nutrients, including high levels of natural antioxidants, *C. vulgaris* has been used in the nutraceutical industries as a replacement for synthetic antioxidants [15], and also in animal feeding as an alternative sustainable high-quality protein feed resource [16]. The growth of the poultry industry increases the demand for agricultural output for feed production. Proteins are the most expensive and most critical components in feed formulation. To address these issues, substantial efforts have been directed towards identifying alternative sustainable high-quality protein feed resources. *C. vulgaris* emerges as a valuable source of protein and other nutrients for animal feeding [17]. Additionally, its abundance of biologically active compounds makes these organisms highly appealing as feed ingredients, offering benefits that extend beyond mere nutrient supply [18]. Moreover, *C. vulgaris* shows potential to mitigate the environmental footprint of poultry production. Studies have shown that incorporating *C. vulgaris* into 10-day-old broilers’ diets can result in enhancing growth performance, gut functional characteristics, and bacterial communities [19]. Moreover, [20] explored the potential of *C. vulgaris* as a candidate alternative to antibiotics in broiler chickens, revealing its influence on affecting the humoral immune response in broiler chicks. The impact of *C. vulgaris* in broilers’ feed on the digestibility and mineral bioaccessibility in meat has been studied in [21], showing an improved nutritional composition of meat in terms of minerals and essential amino acid content.

This study aimed to assess the impact of *C. vulgaris* supplementation in broilers’ diet, alone or in combination with vitamin E, on meat quality parameters, nutritional value, and oxidative stability during storage time.

## 2. Materials and Methods

The experimental trial was carried out with the approval (no. 2761/03.03.2021) of the Ethics Committee of the National Research and Development Institute of Animal Biology and Nutrition in Romania. The experimental procedures followed the guidelines specified in Romanian Law 43/2014 regarding the handling and protection of animals used for experimental purposes and complied with Directive 2010/63/EU on the protection of animals used for scientific purposes.

### 2.1. Experimental Design

An experiment was conducted on 180 COBB 500 broiler chickens (14 days old), assigned into six treatments, following a 2 × 3 factorial arrangement. The initial average weight of birds at 14 days of age was 421.48 g. A corn–soybean meal diet was supplemented with 3 levels of *C. vulgaris* (0% (C1), 1% (E1), 2% (E2)), 2 levels of vitamin E (0% (C1), 250 ppm (C2)), and a combination of the two supplements (1% *C. vulgaris* + 250 ppm vitamin (E3), 2% *C. vulgaris* + 250 ppm vitamin (E4)), as presented in Table 1. Each group included five replicates with six birds per replicate and thirty chicks per treatment. The plant material used in this study consisted of a powder of microalga *Chlorella vulgaris*, supplied by Vivio (Brzozów, Poland).

The chicks were housed in digestibility pens in a poultry experimental hall with controlled environmental conditions (average temperature/total period: 25.08 ± 0.58 °C; humidity: 61.27 ± 4.79%; ventilation/broiler: 0.61 ± 0.16%). The light regimen was 23 h light/1 h darkness. The chicks had free access to feed and water. At the end of the feeding trial, 6 broilers (42 days old) were randomly selected from each treatment group and slaughtered by cervical dislocation. The thigh meat samples without skin were collected to assess the impact of the dietary supplements on the meat quality in terms of proximate composition, vitamin E, fatty acid profile, and oxidative stability parameters. Samples were stored in plastic bags at −80 °C until further analysis. Instrumental color measurements and pH assessment were performed on fresh meat samples.

### 2.2. Determination of Meat Quality Traits

The pH was measured on thigh muscles from the right side of the bird using a glass penetration pH electrode (HI9025, Hanna Instruments, Woonsocket, RI, USA) at 45 min postmortem. The pH value was determined as the average of three replicate measurements taken on the same muscle. The standard color parameters, lightness (L*), redness (a*), and yellowness (b*), were determined for thigh meats with a Minolta CR-400 Chroma Meter (Minolta Camera Co. Ltd., Osaka, Japan). The final CIELAB color parameters were the average of 3 readings, taken from 3 spots at the surface of thigh meats.

The chroma value (C*) was calculated as explained in [22], using the following equation:

C* = (a*^2^ + b*^2^)^1/2^, (1)

where a* = redness and b* = yellowness.

The hue angle (h*) was assessed with the following formula:

h* = tan^−1^(b*/a*) × (360/2π), (2)

where a* = redness and b* = yellowness.

The total color difference (ΔE*) in the thigh meat samples between the control diet (C1) and the experimental diets was calculated according to the following equation:

ΔE* = (ΔL* + Δa* + Δb*)^1/2^, (3)

where L* = lightness, a* = redness, and b* = yellowness.

Cooking loss was assessed as described in [23]. Individual samples of thigh meat with similar weight and size were packed in plastic bags and cooked in a water bath (Memmert, Schwabach, Germany) for 30 min at 85 °C. After cooling at room temperature, the samples were dried with a paper towel and reweighed. The cooking loss was calculated by the following equation:

Cooking loss % = (W1 − W2) × 100/W1,(4)

where W1 = initial sample weight before cooking and W2 = sample weight after cooking.

To evaluate drip loss, samples of fresh thigh muscle were weighed (W1), placed into a plastic bag, and hung in a refrigerator at 4 °C for 24 h [24]. Subsequently, samples were dried and weighed again (W2), using the following equation:

Drip loss (%) = (W1 − W2) × 100/W1,(5)

where W1 = initial sample weight and W2 = final sample weight.

### 2.3. Chemical Analysis

#### 2.3.1. Proximate Composition

The proximate composition of *C. vulgaris* and meat samples was determined using the reference method as described below: crude protein (ISO 5983-2/2009 [25]) with the Kjeldahl method and a semiautomatic Kjeltec auto 1030 Tecator Instruments (Höganäs, Sweden), crude fat (SR ISO 6492/2001 [26]) with the method of continuous solvent extraction and the equipment Soxtec 2055 Foss Tecator (Höganäs, Sweden), crude fiber using the intermediary filtration method and the equipment Fibertec 2010 System Foss Tecator (Höganäs, Sweden), and dry matter (ISO 6496/2001 [27]) and ash (ISO 2171/2010 [28]) using the gravimetric method and a Nabertherm calcination furnace (Nabertherm GmbH, Lilienthal, Germany).

#### 2.3.2. Fatty Acid Determination 

The fatty acid profiling of *C. vulgaris* and thigh meat was assessed as outlined in [29]. Fatty acids (FAs) were converted into FA methyl ester (FAME). Afterward, derivatives of FAs were analyzed using a Perkin Elmer Clarus 500 gas chromatograph (Waltham, MA, USA) equipped with a capillary column with a highly polar stationary phase (Thermo Electron, Waltham, MA, USA), with dimensions of 60 m × 0.25 mm × 0.25 µm film. Detection was conducted using a flame ionization detector (FID), and identification and quantification were achieved by referencing analytical standards.

Lipid quality indexes in thigh meat were determined based on the data obtained from the fatty acid composition of thigh samples, and their relevance to human nutrition was assessed. They were calculated using the following equations [30,31,32,33,34,35]:

AI (Atherogenic Index) = (C12:0 + 4 × C14:0 + C16:0)/(ΣMUFA + Σn6 + Σn3)(6)

TI (Thrombogenic Index) = (C14:0 + C16:0 + C18:0)/(0.5 × ΣMUFA + 0.5 × Σn6 + 3 × Σn3 + Σn3/Σn6)(7)

OFA (hypocholesterolemic fatty acids) = (C14:0 + C16:0) dietary FA with an undesirable effect of increasing cholesterol levels in humans(8)

DFA (hypocholesterolemic fatty acids) = (MUFA + PUFA + C18:0) dietary fatty acids with a beneficial neutral effect on lowering cholesterol levels in humans(9)

HPI (Health-Promoting Index) = UFA/[C12:0 + (4 × C14:0) + C16:0])(10)

PI (Peroxidisability Index) = (monoenoic acid × 0.025) + (dienoic acid × 1) + (trienoic acid × 2) + (tetraenoic acid × 4) + (pentaenoic acid × 6) + (hexaenoic acid × 8)(11)

DBI (Double Bond Index) = (% monoenoic acids) + 2 (% dienoic acids) + 3 (% trienoic acids)/100(12)

IV (Iodine Value) = (0.95 × C16:1) + (0.86 × C18:1n-9) + (1.732 × C18:2n-6) + (2.616 × C18:3n-3) + (0.785 × C20:1).(13)

h/H-hypocholesterolemic FAs/hypercholesterolemic FAs ratio = 5 [C18:1 cis n-9 + C18:2n-6 + C18:3n-6 + C18:3n-3 + C20:3n-6 + C20:4n-6 + C20:5n-3 + C22:4n-6 + C22:5n-3 + C22:6n-3)]/(C14:0 + C16:0).(14)

COX (Calculated oxidizability) = (1 × C18:1 + 10.3 × C18:2 + 21.6 × C18:3)/100(15)

OS (Oxidative susceptibility) = MUFA + 45 × C18:2 + 100 × C18:3(16)

#### 2.3.3. Liposoluble Antioxidants

The liposoluble antioxidants (vitamin E and xanthophylls) were analyzed as previously described in [36]. The extraction procedure involved saponification with ethanolic KOH and extraction with petroleum ether. The final extract was washed with distilled water to remove any alkaline traces and evaporated under vacuum until dry. The concentrated extracts were dissolved in ethanol. 

Xanthophylls were analyzed using an HPLC (Finningan Surveyor Plus, Thermo-Electron Corporation, Waltham, MA, USA) with a PDA-UV detector at wavelength 445 nm, and a C18 reversed-phase column with a stationary phase of 5 µm (250 × 4.60 mm i.d.) (Nucleodur, Macherey-Nagel, Duren, Germany). The chromatographic analysis was performed in isocratic conditions, involving a mobile phase which consists of 87% acetone and 13% water, at a flow rate of 1 mL/min.

Vitamin E was analyzed using an HPLC (Vanquish Thermo-Electron Corporation, Waltham, MA, USA), a PDA-UV detector at wavelength 292 nm, and a HyperSil BDS C18 column, with silica gel, dimensions 250 × 4.6 mm, and particle size 5 µm (Thermo-Electron Corporation, Waltham, MA, USA). The mobile phase used was 96% methanol and 4% water, with a flow rate of 1.5 mL/min, in isocratic conditions.

#### 2.3.4. Water-Soluble Antioxidants

The total polyphenol content (TPC) was measured using the Folin–Ciocalteu spectrophotometric method [30]. A calibration curve of gallic acid was employed to quantify the total phenol content, and the results were expressed as mg of gallic acid equivalents per g of dried sample (mg GAE/g).

The total flavonoid content was determined using the aluminum chloride colorimetric method described in [30]. The absorbance was measured at 410 nm against a blank using a UV-VIS spectrophotometer (Jasco V-530, Japan Servo Co. Ltd., Tokyo, Japan). A calibration curve with quercetin as the standard was used and the flavonoid content was expressed as mg of quercetin equivalent (QE) per g.

#### 2.3.5. Antioxidant Capacity Analysis

The antioxidant capacity of *C. vulgaris* was evaluated using two different spectrophotometric methods for the determination of DPPH and iron chelating ability. A DPPH solution prepared in methanol (0.2 mM) was mixed with the sample extract and distilled water in a ratio of 2:0.4:1.6 (*v*/*v*/*v*). The absorbance was then measured at 517 nm using a spectrophotometer (Jasco V-530, Japan Servo Co., Ltd., Tokyo, Japan). Trolox was used as a reference for standard calibration curves to analyze the DPPH concentration [37]. The results were expressed as mmol of Trolox equivalents per kg of sample (mmol eq Trolox/kg sample).

To assess the chelating effect on ferrous ions, a method described by [38] was used. The method involves measuring the absorbance of the purple complex formed when an extract competes with ferrozine for ferrous ions. This method is based on measuring the absorbance of the purple complex formed when an extract competes with ferrozine for ferrous ions. The absorbance was measured using a UV-VIS spectrophotometer (JASCO V-560, Japan Servo Co. Ltd., Tokyo, Japan) at 562 nm, compared to a blank. 

#### 2.3.6. Oxidative Stability of Meat

The oxidative stability of the thigh meat samples was assessed by evaluating primary lipid degradation parameters, including peroxide values (PVs), conjugated dienes (CDs), and conjugated trienes (CTs), as well as secondary parameters such as p-anisidine values and thiobarbituric acid-reactive substances (TBARSs). These measurements were conducted using previously described methods [39].

Additionally, markers of lipid peroxidation included myoglobin derivatives (metmyoglobin, deoxymyoglobin, and oxymyoglobin). All the aforementioned parameters were measured spectrophotometrically using a V-530 Jasco spectrophotometer (Tokyo, Japan), following the methods described in [39].

### 2.4. Relative Prevention of Lipid Oxidation—Efficiency Factor 

The relative inhibition of lipid oxidation was quantified as described in [40], as an efficacy ratio on day 7 of storage, calculated by dividing the PV or TBARS value of the control group by that of the experimental groups. A higher efficiency factor indicates greater effectiveness of the dietary treatments in decreasing lipid oxidation (PV or TBARS) in the thigh meat of broilers.

### 2.5. Statistical Analysis

The analytical data regarding the nutritional composition, lipid quality indexes, and markers of lipid peroxidation assessed in thigh meat samples were analyzed using a 2-way ANOVA, followed by Tukey’s HSD test, with XLStat (Addinsoft, New York, NY, USA). The statistical model included the fixed effects of vitamin E (0 and 250 ppm) and *C. vulgaris* (0%, 1%, and 2%) as well as the interaction between them. *p*-values less than 0.05 were considered significant. Each broiler that was chosen for collecting samples of thigh meat was treated as an experimental unit.

## 3. Results

### 3.1. Nutritional Composition of C. vulgaris

The results regarding the nutritional composition of *Chlorella vulgaris* are presented in Table 2.

*C. vulgaris* was found to be a rich source of essential nutrients, including proteins, fatty acids, vitamins, and antioxidants. The nutritional composition analysis highlighted the high protein content of the analyzed microalga, making it a valuable source of high-protein nutritional supplements. The antioxidant analysis of *C. vulgaris* showed elevated concentrations of vitamin E and xanthophylls, which increase the antioxidant capacity and enhance the nutraceutical potential of *C. vulgaris*. 

The FA profile of *Chlorella vulgaris* allowed for the identification of 14 FAs (Table 2), including saturated (C12:0, C14:0, C15:0, C16:0, C17:0, C18:0 and C20:0), monounsaturated (C14:1, C15:1, C16:1, C17:1 and C18:1), and polyunsaturated (C18:2, C18:3) FAs. The primary fatty acids of *Chlorella* were linoleic, palmitic, alpha-linolenic, oleic, heptadecenoic, and heptadecanoic acids.

### 3.2. Meat Quality Traits

The effect of dietary treatments on the meat quality traits of broilers is shown in Table 3. Thighs from birds fed with diets supplemented with *C. vulgaris* and vitamin E (E3 and E4 groups) had significantly higher values of pH than birds from the control group (C1). Thigh lightness (L*) registered a slight increase in the E3 and E4 groups compared to the control, but the values were not statistically different. 

Dietary incorporation of *C. vulgaris*, including those supplemented with vitamin E, resulted in a minor but statistically significant (*p* < 0.05) increase in b*, C*, and h*, while a* registered lowered values (*p* < 0.05) when compared to the control group. Nevertheless, the calculated color differences (ΔE*) between the samples of the studied groups showed a minor variation, which led to the conclusion that no differences in color could be observed. Neither cooking loss nor drip loss was affected by dietary treatments (*p* > 0.05) in thigh meat. 

### 3.3. Proximate Composition of Meat

**Figure 1:**
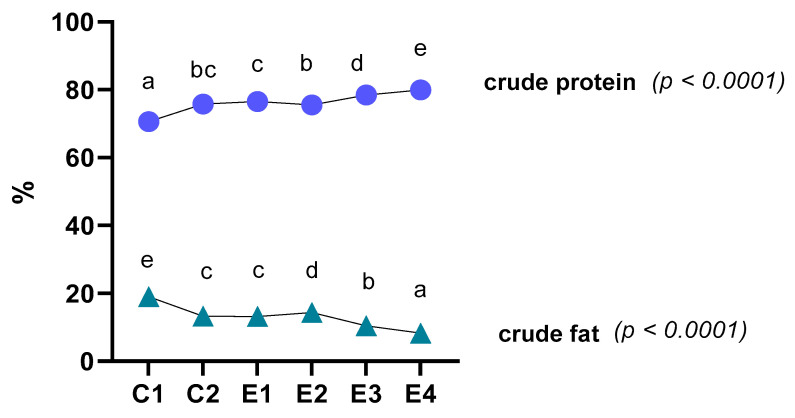
Effect of dietary treatments on the levels of crude protein and crude fat of thigh muscle of broilers. Different letters indicate a significant difference (*p* < 0.05).

The effect of dietary microalgae on the proximate composition of thigh meat is presented in Figure 1. The incorporation of 1 and 2% *C. vulgaris*, alone or with vitamin E, resulted in an increase in protein content in the meat (*p* < 0.0001). Ash content was increased in all the groups, although no significant differences were found between them, except for the 2% *C. vulgaris* and vitamin E group (E4). Fat content was significantly lower (*p* < 0.0001) in the thigh muscle of broilers that were fed a diet with *C. vulgaris* or vitamin E or a combination of them, in comparison to those fed the control diet.

### 3.4. Fatty Acid Profiling of Meat

The effect of *C. vulgaris* incorporation, alone or combined with vitamin E, on the fatty acid composition of thigh meats is presented in Table 4. Dietary treatments induced changes in the fatty acid profile of thigh meat. The percentage of palmitic acid was lower (*p* < 0.0001) in the thighs of broilers fed *C. vulgaris* and *C. vulgaris* + vitamin E diets. Chlorella-fed broilers had a higher concentration of α-linolenic, erucic, and eicosatrienoic acids (*p* < 0.0001) in their thigh meat compared to the control group. Linolenic acid was detected in a small amount only in groups supplemented with *C. vulgaris* and vitamin E (E3 and E4 groups). The proportions of docosapentaenoic acid (DPA) and docosahexaenoic acid (DHA) were enhanced in the thighs of broilers fed with *C. vulgaris*, with significant increases being observed in the group supplemented with 2% *C. vulgaris* and vitamin E (E4). In fact, both DPA and DHA levels increased by 2.01-fold and by 1.60-fold in the 2% *C. vulgaris* + vitamin E group (E4). Moreover, the total amount of omega-3 FAs was higher in the thighs of broilers fed with *C. vulgaris*.

Concerning the fatty acid ratios, a significantly decreased n-6/n-3 ratio (*p* < 0.05) in the thigh muscle was observed in groups supplemented with 2% *C. vulgaris*, alone or with vitamin E (E2 and E4 groups), and also in the 1% *C. vulgaris* with vitamin E group (E3), when compared with the control. The PUFA/SFA ratio was increased across all dietary treatments (*p* < 0.0001). Both DFAs and OFAs were influenced by the *C. vulgaris* supplementation. The highest percentage of hypocholesterolemic fatty acids (DFAs) and the lowest of hypercholesterolemic fatty acids (OFAs) were shown for thighs from the *C. vulgaris* supplementation groups. The DFA/OFA index registered the highest increase in the 2% *C. vulgaris* with vitamin E group (E4).

### 3.5. Lipid Quality Indexes

To estimate the lipid nutritional and health benefits of *C. vulgaris*, alone or combined with vitamin E, the lipid quality indexes were calculated (Table 5). The values of oxidative susceptibility (OS), double-bond index (DBI), iodine value (IV), and peroxidisability index (PI) from meat samples of experimental groups presented a significant (*p* < 0.05) increase compared to the control group. A decreasing tendency was observed in the AI of the thighs from broilers fed with *C. vulgaris*, a significant reduction (*p* < 0.05) being observed in the groups with 2% *C. vulgaris* supplementation (E2 and E4). In the same manner, the TI registered the highest significant (*p* < 0.05) decrease as an effect of the 2% *C. vulgaris* supplementation in broilers’ diet (E2 and E4). The h/H values were significantly higher in *C. vulgaris*-supplemented groups when compared to the two control groups. The calculated oxidizability value (COX) was also measured for all meat samples. The results showed a significant increase (*p* < 0.05) in the COX value in all the groups, except the E3 group where the differences were not significantly higher. Therefore, the thigh meat from the experimental groups (including C1 and excluding E3) exhibits better oxidative stability and thus has a longer shelf life. 

The health-promoting index (HPI) enables the assessment of the impact of fatty acids on cardiovascular diseases. It represents the inverse of the atherogenicity index, indicating inverse values compared to the AI, and, in the present study, registered a favorable increase in the groups with supplemented *C. vulgaris*. The significant variation in values obtained from the different indexes highlights their distinct meanings and the necessity of using each index under specific conditions.

### 3.6. Vitamin E Concentration in Thigh Meat

The effect of feeding treatments on the thigh meat content of vitamin E is presented in Table 6. The concentration of vitamin E was influenced by feeding treatments (*p* < 0.05), showing a significant increase in all the groups compared to the control group (C1); the increase ranged between 21.7 and 44.69%. Supplementation of broiler diet with 2% *C. vulgaris* and 250 mg/kg vitamin E exhibited a 1.45-fold increase in vitamin E concentration in thigh meat compared to the control group, being the highest level registered in thigh meat in this experiment.

### 3.7. Oxidative Stability of Meat

Supplementation of broilers’ diets with *C. vulgaris*, alone or combined with vitamin E, had no significant effect on the primary oxidation products in thigh meat stored for 7 days in refrigerated conditions (Table 7), although a decreasing tendency was observed for conjugated dienes, conjugated trienes, and peroxide value in the experimental groups compared to the control group (C1). Regarding the secondary oxidation products, TBARS levels were significantly reduced (*p* < 0.05) in the experimental groups relative to the reference group (C1) after 7 days of refrigeration. For p-anisidine, lower concentrations were also observed in the experimental groups, but without statistical significance. 

**Figure 2:**
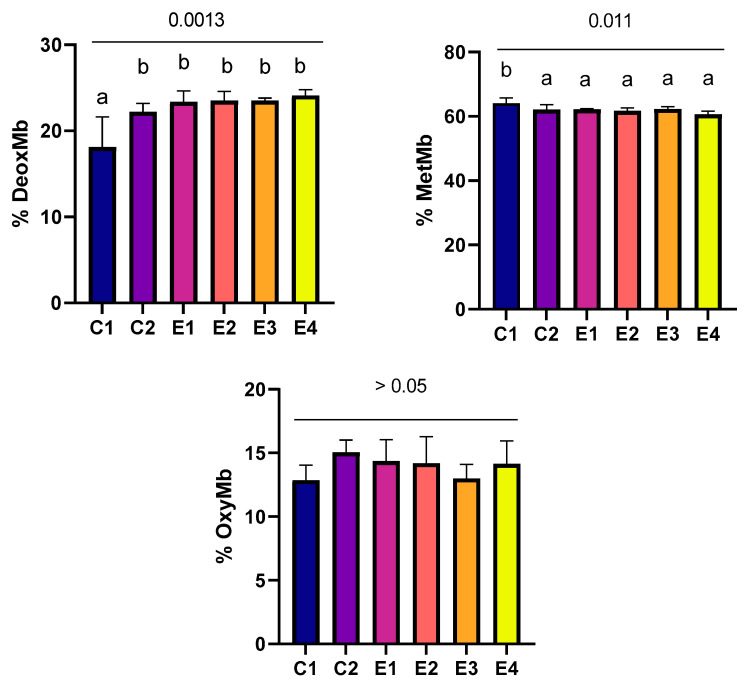
Concentrations of myoglobin derivatives in thigh meat. Data represented as means ± standard deviation (SD); means with no common superscript are significantly different (*p* < 0.05).

Incorporation of *C. vulgaris*, alone or combined with vitamin E, in broilers’ diet led to changes in the hem pigments in thigh meat stored for 7 days in refrigerated conditions (Figure 2). The results showed a significant increase (*p* > 0.05) in the deoxymyoglobin percentage in all the experimental groups compared to the control group, with no significant differences observed between the experimental groups. Another positive effect was the decrease in metmyoglobin concentrations in the thigh meat of the experimental groups, with significant differences noted (*p* < 0.05) when compared to the control group. The oxymyoglobin levels did not register any significant differences between the studied groups.

**Figure 3:**
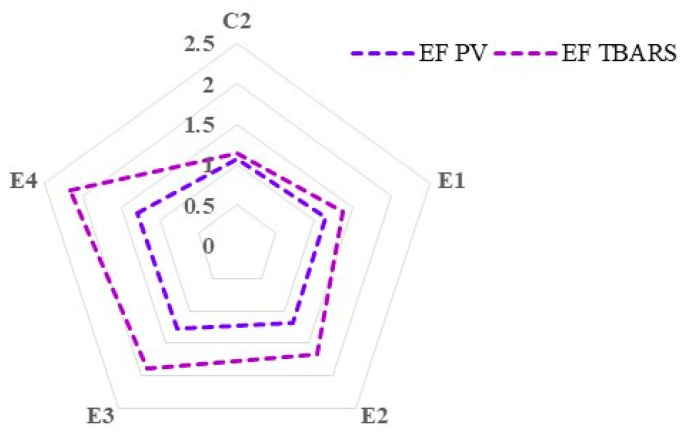
Relative prevention of lipid oxidation expressed as the efficiency factor (EF) from thigh meat samples on day 7 of refrigerated storage, calculated as the ability of the contained antioxidants to inhibit lipid oxidation, measured as peroxide value (PV) and thiobarbituric acid reactive substances (TBARS) in thigh meat sample.

Figure 3 shows the efficiency factor of the dietary treatments in relation to PV and TBARS measurements on day 7 of refrigerated storage of thigh meat. A higher efficiency factor indicates a greater ability of the antioxidants to reduce lipid oxidation (as measured by PV or TBARS) in the meat samples.

After 7 days of storage in refrigerated conditions, thigh meat from the group receiving a diet supplemented with 2% *C. vulgaris* and vitamin E (E4) showed the highest efficiency factor (lowest degree of lipid oxidation compared to the control group C1), followed by the group fed a diet with 1% *C. vulgaris* and vitamin E (E3), for both PV and TBARS measurements. The inclusion of *C. vulgaris* (1% and 2%) in combination with vitamin E (250 mg/kg) in broiler diets exhibited the best prevention of lipid oxidation after 7 days of refrigerated storage, defined by the highest efficiency factors assessed in terms of secondary oxidation products.

## 4. Discussion

### 4.1. Nutritional Composition of C. vulgaris

*C. vulgaris* is considered a promising feed source due to its high growth rate and rich content of essential nutrients, particularly amino acids, fatty acids, and antioxidants. *C. vulgaris* has been used for partial dietary replacement of conventional protein sources like soybean meal, to enhance sustainability and lower the environmental impact of animal production [41]. In this study, a high protein level was assessed (around 50% DM), which was in line with the previously reported data [3]. The predominant fatty acids in *C. vulgaris* are the essential PUFAs 18:2n-6 and 18:3n-3, which have recognized positive effects on health for both humans and animals. *C. vulgaris* contains large amounts of various pigments, including chlorophylls a and b, as well as carotenoids such as β-carotene and lutein [8]. The total carotenoid content in *C. vulgaris* can vary, with levels reaching up to 3.49 g/kg DM. In this study, only the xanthophylls were assessed from the total carotenoids, since lutein is considered the predominant carotenoid in *C. vulgaris* [42]. A xanthophyll concentration of 0.98 g/kg DM was analyzed, which was below the concentration of total carotenoid content reported previously. It was shown that different carotenoid concentrations may be observed depending on the drying process, culturing conditions, and harvest time [43]. Additionally, the analytical results of this study have shown that *C. vulgaris* is a valuable source of vitamin E, as was also reported in [44]. These compounds possess significant antioxidant and radical scavenging properties [10].

### 4.2. Meat Quality Traits

Meat color is essential for the meat industry because it significantly shapes consumers’ perceptions of product quality and influences their purchasing decisions. In meat and meat products, color is a key parameter that consumers associate with quality, particularly in fresh meat products [45]. An elevated yellow color of thigh meat is preferred by consumers from various regions around the globe [46].

In the present study, dietary incorporation of *C. vulgaris* resulted in a minor increase in b*, C*, and h*, while a* registered a slight decrease when compared to the control group. The low L* values observed in the present study were comparable with the ones reported in [47], which registered an L* value of 32.53 and a pH of 6.62, in line with the results of the current study. Lower redness values are usually linked to higher concentrations of MetMb. Nevertheless, no differences in MetMb levels were observed between the groups that received dietary supplements, which can be explained by the minor variation in the color differences (ΔE*) calculated between the samples of the studied groups. A study conducted in [48] reported that color changes measured instrumentally are considered visible if ∆E > 2. In this study, the supplementation of broiler diets with 1% or 2% *C. vulgaris*, alone or in combination with vitamin E, resulted in no visible change of color, with a maximum ∆E of 1.72 observed in the E3 group. In contrast to the present study, [16] reported a yellower breast muscle (*p* < 0.0001) after the incorporation of a higher proportion of *C. vulgaris* (10%) in broilers’ diet compared to the present study (1% and 2% *C. vulgaris*), while the redness decreased with the addition of *C. vulgaris* in higher concentrations.

The postmortem pH value significantly affects color stability. Immediately after slaughter, meat has a pH of around 7, and it drops to approximately 5.5–5.8 due to glycolysis, increasing the likelihood of autoxidation [49]. In this study, pH registered higher values in meat from the experimental groups, with statistical significance only in groups that received *C. vulgaris* with vitamin E (E3 and E4 groups). Drip and cooking loss were not significantly changed by the 1% and 2% *C. vulgaris* inclusion levels, with or without vitamin E, but a numerical reduction in cooking loss was observed in groups fed *C. vulgaris* with vitamin E (E3 and E4 groups). In line with these results, [16] also reported no significant changes in drip loss and cooking loss in breast meat after 10 and 15% *C. vulgaris* dietary supplementation in broilers’ diets. 

### 4.3. Proximate Composition of Meat

The inclusion of *C. vulgaris* in poultry diets has demonstrated a significant increase (*p* < 0.05) in the protein concentration of thigh meat, simultaneously with a significant decrease in the crude fat of thigh meat. This enhancement is thought to be attributed to Chlorella’s abundant amino acid profile and bioactive compounds, which support muscle growth and protein synthesis mechanisms. Similar results were reported by [21] who observed an increase in protein content in breast meat as a consequence of 15 and 20% incorporation of *C. vulgaris* in broiler’s diet. The same authors assumed that the increased protein content could be linked to the greater availability of amino acids from Chlorella proteins, enhanced nutrient absorption, or a combination of both. In another study [50], it was shown that an increase in the villus heights and crypt depths and, consequently, increased absorption area can be observed when supplementing broiler diets with 1% Chlorella by-products. Moreover, it has been reported that the inclusion of Chlorella in broilers’ diet can increase Lactobacillus spp. in broilers’ intestines, thus enhancing digestion and nutrient absorption [51]. In addition, it has been proposed that DHA supplementation enhances muscle protein synthesis in growing pigs [52], and can reduce lipid content in pig muscle [53]. This is consistent with the results of the current study, which showed significantly higher DHA levels in the thigh meat of the experimental groups fed *C. vulgaris*, compared to the control group.

The results of this study have shown a significantly lower fat content in the thigh muscle of broilers in the experimental groups compared to the control group, which was also reported by [21] after incorporation of 15 and 20% *C. vulgaris* in the broilers’ diet. A lower fat content in muscle can be attributed to dietary changes in fatty acids. Diets enriched with n-3 PUFAs are linked to decreased fat deposition [54]. In the current study, data regarding the fatty acid profile of *C. vulgaris* show that this microalga is a rich source of n-3 PUFAs, as [55] also reported.

### 4.4. Fatty Acid Profiling of Meat

The minor variations registered in SFA and MUFA concentrations in the thigh meat of broilers fed with diets supplemented with *C. vulgaris*, alone or with vitamin E, compared to the control group, were consistent with previously published reports [56]. In contrast, the concentrations of n-3 FA, PUFAs, and arachidonic acid increased in the thigh meat of broiler chickens that received *C. vulgaris*, when compared to the control group, results that align with previous findings in broiler chickens [57,58]. In this study, the DHA level registered a slight increase in the experimental groups, being significantly higher (*p* < 0.05) in the group that received 2% *C. vulgaris* and vitamin E. 

*C. vulgaris* is a microalga that contains high levels of methionine and lysine. The authors of [59] suggested that the high level of n-3 fatty acids in the meat of broilers that received a diet supplemented with microalgae may be linked to its high methionine and lysine contents. The authors of [60] have shown that methionine and lysine can enhance the concentrations of n-3 fatty acids in broiler meat. Alongside the changes observed in the fatty acid profile, in this study, a significant reduction in total fat content was also noted in the thigh of broilers fed *C. vulgaris*. Methionine functions as a lipotropic agent by serving as a methyl donor for phospholipid synthesis and acting as a precursor for lipoproteins essential for lipid transport from the liver [61]. Lipid accumulation in tissues is mainly linked to lipid synthesis and breakdown, indicating that dietary methionine influences lipid metabolism in broilers.

The proportion of hypocholesterolemic to hypercholesterolemic fatty acids (DFA/OFA indexes) reveals the influence of particular fatty acids on cholesterol metabolism. Higher DFA/OFA values are deemed more favorable for human health [62]. The DFA/OFA indexes obtained in the current study ranged from 3.799 to 4.038 for the thigh meat of broilers that received diets with *C. vulgaris*, being higher compared to the control group which registered a value of 3.756.

### 4.5. Lipid Quality Indexes

AI and TI, which assess the impact of individual fatty acids on the likelihood of atheroma and thrombus formation, are additional markers relevant to consumer health. An AI above 1.0 is considered potentially harmful to health [63]. To our knowledge, there are no studies on the effects of a *C. vulgaris* supplementation in broilers’ diet on atherogenic and thrombogenic activities. The results of AI in the thigh meat of broilers obtained in the present study were beneficial for human health, regardless of dietary treatments applied. Nevertheless, the lowest significant values (*p* < 0.05) were noted in the meat from groups receiving 2% *C. vulgaris* (E2 and E4 groups). Similarly, significantly reduced TI values (*p* < 0.05) were observed in the thigh meat of broilers from the same experimental groups (E2 and E4).

The PI indicates the sensitivity of PUFAs to oxidation. Elevated PI levels correlate with increased fatty acid oxidation. Nevertheless, high PI levels resulting from substantial omega-3 and omega-6 PUFAs contribute to enhanced antioxidant and anti-inflammatory effects [64]. As expected, in the current study, the PI levels were increased, with significantly higher values being observed in the group with 2% *C. vulgaris* and vitamin E when compared to the control group. Moreover, the results showed that the DBI, IV, and Cox values were higher in the experimental groups, with significantly increased values when 2% *C. vulgaris* was included in the broilers’ diet (E2 and E4 groups). On the contrary, HPI and h/H registered the highest values as a result of 2% *C. vulgaris* supplementation, being linked with a positive effect in lowering cholesterol levels. These findings highlight the nutraceutical value of fatty acids from *C. vulgaris*, in agreement with the outcomes reported in [65].

### 4.6. Vitamin E Assessment in Meat

Vitamin E concentrations increased significantly (*p* < 0.05) in all the groups compared to the control group (C1), with the highest value being registered in the meat of broilers that received 2% *C. vulgaris* and vitamin E (E4 group). There is a notable lack of research on the impact of incorporating *C. vulgaris* into broiler diets on meat vitamin E levels. A study conducted in [55] investigated the effects of 10% *C. vulgaris* in broiler diets, alone or combined with exogenous CAZymes, on meat quality. Unlike our results, the dietary treatments did not affect vitamin E in breast meat. Moreover, in the thigh, the dietary supplementation with *C. vulgaris*, with and without exogenous CAZymes, decreased the levels of α-tocopherol and γ-tocopherol relative to the control. Supplementation of diets with high levels of *C. vulgaris* can lead to high amounts of carotenoids in meat [44,55], which can interfere with the absorption of vitamin E. Previous research indicated that α-tocopherol competes for absorption with other lipid micronutrients such as γ-tocopherol, carotenoids, and vitamins A, D, and K. These competitive interactions are probably caused by common uptake pathways [66]. The findings of the current study highlight that the inclusion of 1% and 2% *C. vulgaris* in broiler diets, alone or with vitamin E, enhanced the vitamin E content of chicken thighs, thereby providing additional benefits for consumers. Vitamin E plays a role in preventing lipid peroxidation by scavenging lipid peroxyl radicals, which otherwise propagate lipid peroxidation [67]. Its antioxidant capacity hinges on its ability to donate phenolic hydrogen to free radicals. Studies have demonstrated that supplementing the diet with vitamin E leads to an accumulation of α-tocopherol in muscle tissue, which delays the oxidation of deoxymyoglobin to metmyoglobin and consequently enhances color stability [68]. 

### 4.7. Oxidative Stability of Meat

Increasing α-tocopherol concentrations in thigh meat can significantly improve its antioxidant stability. Numerous studies have demonstrated that dietary supplementation with vitamin E reduces muscle membrane susceptibility to Fe^2+^-induced lipid oxidation [69,70].

Data regarding the markers of lipid oxidation in thigh meat revealed a significant decrease in TBARSs in the thigh tissues of birds supplemented with *C. vulgaris* compared to the control. This finding aligns with previous research highlighting the strong antioxidant capacity of Chlorella in broiler chickens [59]. TBARS levels exceeding 0.5 mg of malondialdehyde per kilogram of fresh meat are considered critical, as consumers can easily detect rancid off-flavors at this degree of lipid oxidation [71]. In the current study, TBARS levels were under this threshold in all the studied groups after 7 days of storage, with the highest TBARS concentration of 0.37 mg/kg registered in the thigh meat of the control group. Research on *C. vulgaris* supplementation in broiler chicks revealed that *Chlorella* increased total white blood cell count and hemoglobin levels and decreased the serum malondialdehyde concentration, further supporting its antioxidant properties [72]. Broilers fed with 20 g/kg *Chlorella* by-products exhibited increased serum superoxide dismutase activity and decreased serum malondialdehyde concentration, suggesting an improvement in the antioxidant status of broilers [50]. Dietary supplementation of Se-enriched *Chlorella* in broiler diets improved the oxidative stability of chicken meat during storage in a refrigerator, as indicated by reduced malondialdehyde values in breast meat after storage [73].

Lipid oxidation is directly linked to pigment oxidation. Deoxymyoglobin (DeoxyMb) is the purple pigment seen in freshly cut meat. After a few minutes of exposure to air, DeoxyMb becomes oxygenated, transforming into oxymyoglobin (OxyMb), which imparts the characteristic bright, cherry-red color. After several hours to days of air exposure, OxyMb converts to metmyoglobin (MetMb), where a water molecule replaces an oxygen molecule, resulting in brown pigmentation. Both DeoxyMb and OxyMb are heme proteins with iron in the ferrous (Fe^+2^) form, whereas MetMb contains iron in the ferric (Fe^+3^) form. The conversion from the ferrous to ferric form occurs due to oxidation [74]. In this study, the metmyoglobin percentage was positively influenced by the dietary treatments, showing significantly (*p* = 0.011) lower values in the experimental groups compared to the control group (C1). Moreover, there was a significant increase in the deoxymyoglobin percentage in all the experimental groups when referred to the control group (C1). On the other hand, no significant differences were found in the relative concentration of oxymyoglobin between the studied groups. Supplementation of diets with vitamin E has been shown to enhance meat quality by decreasing lipid oxidation in skeletal muscle. This, in turn, positively impacts meat discoloration by delaying the oxidation of myoglobin or oxymyoglobin to metmyoglobin, which leads to a brown color that is unappealing to consumers [75]. In this study, supplementation of broilers’ diet with *C. vulgaris* led to a decrease in redness, contrasting with the increasing effect on yellowness in thigh muscles. Generally, lower redness values are linked to higher concentrations of metmyoglobin. Nevertheless, in the current study, lower metmyoglobin levels were registered in the experimental groups. Beyond the impact of lipid peroxidation on meat flavor, color, and texture, lipid peroxidation poses a health risk when meat that has undergone this process is consumed, due to the autoxidation of unsaturated lipids and cholesterol that leads to the formation of atherogenic compounds [76].

## 5. Conclusions

The results from the current study demonstrate that *C. vulgaris* incorporation in broilers’ diet can enrich chicken meat with n-3 FA and vitamin E. Moreover, it can be considered an effective and practical approach to not only enhance the nutritional value and health benefits of meat lipids by decreasing the n-6/n-3 PUFA ratio but also to delay the meat’s susceptibility to lipid oxidation. 

The current results may recommend supplementation with 2% *Chlorella vulgaris* and 250 ppm vitamin E in broiler chickens’ diet for meat quality improvement in terms of proximate composition, lipid quality, antioxidant status, and oxidative stability during storage in refrigerated conditions.

## Figures and Tables

**Table 1:** The structure of the experimental diets.

	Grower Stage (11–22 Days)						Finisher Stage (23–42 Days)					
Ingredients	C1	C2	E1	E2	E3	E4	C1	C2	E1	E2	E3	E4
Corn	42.00	42.00	42.00	42.00	42.00	42.00	42.00	42.00	42.00	42.00	42.00	42.00
Wheat	18.73	18.73	19.66	20.59	19.66	20.59	20.56	20.56	21.49	22.42	21.49	22.42
SBM	30.54	30.54	28.79	27.05	28.79	27.05	28.19	28.19	26.45	24.71	26.45	24.71
Oil	4.10	4.10	3.79	3.47	3.79	3.47	5.11	5.11	4.79	4.48	4.79	4.48
*C. vulgaris*	0	0	1.00	2.00	1.00	2.00	0	0	1	2	1	2
Lysine	0.19	0.19	0.25	0.31	0.25	0.31	0.09	0.09	0.15	0.21	0.15	0.21
Methionine	0.24	0.24	0.26	0.28	0.26	0.28	0.20	0.20	0.21	0.23	0.21	0.23
Threonine	0.03	0.03	0.06	0.09	0.06	0.09	-	-	0.03	0.06	0.03	0.06
CaCO_3_	1.29	1.29	1.30	1.31	1.30	1.31	1.17	1.17	1.18	1.19	1.18	1.19
Ca(H_2_PO_4_)_2_	1.48	1.48	1.48	1.49	1.48	1.49	1.30	1.30	1.31	1.32	1.31	1.32
Salt	0.36	0.36	0.35	0.36	0.35	0.36	0.33	0.33	0.33	0.33	0.33	0.33
Choline	0.04	0.04	0.05	0.05	0.05	0.05	0.05	0.05	0.05	0.05	0.05	0.05
Premix	1	1 *	1	1	1 *	1 *	1	1 *	1	1	1 *	1 *
Total	100	100	100	100	100	100	100	100	100	100	100	100
Calculated analysis, %												
Dry matter	88.30	88.30	88.41	88.51	88.41	88.51	88.38	88.38	88.48	88.59	88.48	88.59
M.E. poultry, (kcal/kg)	3086	3086	3086	3086	3086	3086	3167	3167	3167	3167	3167	3167
Crude protein	20	20	20	20	20	20	19	19	19	19	19	19
Crude fat	5.93	5.93	5.69	5.45	5.69	5.45	6.92	6.92	6.68	6.44	6.68	6.44
Crude fiber	3.78	3.78	3.84	3.90	3.84	3.90	3.71	3.71	3.77	3.83	3.77	3.83
Calcium	0.84	0.84	0.84	0.84	0.84	0.84	0.76	0.76	0.76	0.76	0.76	0.76
Available phosphorous	0.42	0.42	0.42	0.42	0.42	0.42	0.38	0.38	0.38	0.38	0.38	0.38
Lysine	1.19	1.19	1.19	1.19	1.19	1.19	1.05	1.05	1.05	1.05	1.05	1.05
Methionine	0.55	0.55	0.56	0.57	0.56	0.57	0.49	0.49	0.50	0.51	0.50	0.51
Met + cis	0.80	0.80	0.89	0.89	0.89	0.89	0.82	0.82	0.82	0.74	0.82	0.74
Threonine	0.78	0.78	0.78	0.78	0.78	0.78	0.71	0.71	0.71	0.71	0.71	0.71
Triphtopan	0.23	0.23	0.22	0.21	0.22	0.21	0.21	0.21	0.21	0.20	0.21	0.20

Premix composition per kg feed: 11.000 IU/kg vitamin A; 2.000 IU/kg vitamin D3; 27 IU/kg vitamin E; 3 mg/kg vitamin K; 2 mg/kg vitamin B1; 4 mg/kg vitamin B2; 14.85 mg/kg pantothenic acid; 27 mg/kg nicotinic acid; 3 mg/kg vitamin B6; 0.04 mg/kg vitamin B7; 1 mg/kg vitamin B9; 0.018 mg/kg vitamin B12; 20 mg/kg vitamin C; 80 mg/kg Mn; 80 mg/kg Fe; 5 mg/kg Cu; 0.60 mg/kg Zn; 0.37 mg/kg Co; 1.52 mg/kg I; 0.18 mg/kg Se. SBM—soybean meal; CaCO_3_—calcium carbonate; Ca(H_2_PO_4_)_2_—monocalcium phosphate; ME—metabolizable energy; Ca—calcium; Av. P—available phosphorus. * Premix was supplemented with 250 mg/kg of vitamin E.

**Table 2:** Nutritional composition of *C. vulgaris*.

Proximate Composition		
DM, %		94.94
CP, %		51.58
EEs, %		3.062
Ash, %		0.861
Antioxidants		
Vitamin E, µg/g		972.5
Total polyphenols, mg equiv. gallic acid/g		1.273
Total flavonoids, mg/g		3.284
Xanthophylls, µg/g		983.4
Antioxidant capacity		
Iron chelating ability, equiv. mg EDTA/g		63.04
DPPH, mmol eq Trolox/kg		12.75
Fatty acids, g/100 g Total FAs		
Lauric acid	C12:0	0.841
Miristic acid	C14:0	0.273
Miristoleic acid	C14:1	0.154
Pentadecanoic acid	C15:0	0.180
Pentadecenoic acid	C15:1	1.080
Palmitic acid	C16:0	23.32
Palmitoleic acid	C16:1	1.492
Heptadecanoic acid	C17:0	6.481
Heptadecenoic acid	C17:1	7.063
Stearic acid	C18:0	1.094
Oleic acid	C18:1	9.330
Linoleic acid	C18:2n6	34.24
Arachidic acid	C20:0	0.000
α Linolenic acid	C18:3n3	14.14
Σ SFAs		32.19
Σ MUFAs		19.11
Σ PUFAs		48.38
PUFAs/SFAs		1.503
Σ Ω3		14.14
Σ Ω6		34.24
Ω6/Ω3		2.422

DM, dry matter; CP, crude protein; EEs, ether extractives; SFAs, saturated fatty acids; MUFAs, monounsaturated fatty acids; PUFAs, polyunsaturated fatty acids. The relative concentration of each fatty acid is reported as a gram of fatty acids/100 g of total fatty acids.

**Table 3:** Effects of dietary treatments on the physicochemical properties of thigh muscle of broiler chickens.

Vit E	*C. vulgaris*	pH 45 min	DL, %	CL, %	L*	a*	b*	C*	h*	ΔE*
0	0	6.398 ^a^	2.013 ^a^	19.25 ^a^	32.94 ^a^	1.295 ^b^	3.392 ^a^	3.464 ^ab^	69.13 ^a^	-
	1	6.505 ^ab^	1.989 ^a^	18.72 ^a^	32.03 ^a^	1.163 ^ab^	3.494 ^a^	3.639 ^ab^	71.75 ^a^	1.070
	2	6.505 ^ab^	1.868 ^a^	18.28 ^a^	32.26 ^a^	1.237 ^ab^	3.842 ^ab^	3.986 ^abc^	72.57 ^a^	1.090
250	0	6.553 ^ab^	2.030 ^a^	19.75 ^a^	32.62 ^a^	1.351 ^b^	3.250 ^a^	3.404 ^a^	67.84 ^a^	1.032
	1	6.743 ^b^	1.977 ^a^	17.81 ^a^	34.03 ^a^	0.751 ^a^	4.731 ^b^	4.230 ^bc^	80.97 ^b^	1.724
	2	6.805 ^b^	1.882 ^a^	17.87 ^a^	34.14 ^a^	0.748 ^a^	4.418 ^b^	4.448 ^c^	80.09 ^b^	1.665
Main effects										
Vit E										
0		6.469 ^a^	1.957 ^a^	18.75 ^a^	32.41 ^a^	1.232 ^b^	3.576 ^a^	3.696 ^a^	71.15 ^a^	-
250		6.701 ^b^	1.963 ^a^	18.48 ^a^	33.59 ^b^	0.950 ^a^	4.133 ^b^	4.027 ^b^	76.30 ^b^	-
*C. vulgaris*										
0		6.476 ^a^	2.021 ^a^	19.50 ^a^	32.78 ^a^	1.323 ^b^	3.321 ^a^	3.434 ^a^	68.48 ^a^	-
1		6.624 ^a^	1.983 ^a^	18.27 ^a^	33.03 ^a^	0.957 ^a^	4.113 ^b^	3.934 ^b^	76.36 ^b^	-
2		6.655 ^a^	1.875 ^a^	18.08 ^a^	33.20 ^a^	0.993 ^a^	4.130 ^b^	4.217 ^b^	76.33 ^b^	-
*p*-Value										
Vit E		0.001	0.945	0.782	0.013	0.005	0.003	0.037	0.001	-
*C. vulgaris*		0.046	0.378	0.447	0.743	0.005	0.001	0.001	0.0001	-
Vit E × *C. vulgaris*		0.616	0.989	0.839	0.075	0.049	0.012	0.194	0.029	-
SEM										
Vit E		0.042	0.062	0.693	0.318	0.066	0.124	0.107	1.073	-
*C. vulgaris*		0.052	0.076	0.849	0.389	0.081	0.152	0.131	0.984	-
Vit E × *C. vulgaris*		0.073	0.107	1.200	0.550	0.114	0.215	0.185	1.880	-

SEM, standard error of the means; DL, drip loss; CL, cooking loss; L*, lightness; a*, redness; b*, yellowness; C*, chroma; h*, hue angle; ΔE*, total color difference. Means in columns followed by the same letter are not significantly different at the 5% level of probability (*p* < 0.05).

**Table 4:** Fatty acid profile in thigh meat.

Fatty Acids	C:D	C1	C2	E1	E2	E3	E4	SEM	*p*-Value
Caproic acid	C6:0	0.062 ^b^	0.027 ^ab^	0.034 ^ab^	0.032 ^ab^	0.040 ^ab^	0.022 ^a^	0.004	0.031
Caprylic acid	C8:0	0.044 ^a^	0.019 ^a^	0.024 ^a^	0.030 ^a^	0.048 ^a^	0.027 ^a^	0.004	0.220
Capric acid	C10:0	0.027 ^a^	0.040 ^a^	0.036 ^a^	0.038 ^a^	0.040 ^a^	0.036 ^a^	0.002	0.154
Lauric acid	C12:0	0.072 ^a^	0.115 ^a^	0.113 ^a^	0.064 ^a^	0.070 ^a^	0.103 ^a^	0.006	0.024
Miristic acid	C14:0	0.456 ^a^	0.522 ^b^	0.488 ^ab^	0.494 ^ab^	0.514 ^b^	0.494 ^ab^	0.006	0.007
Miristoleic acid	C14:1	0.077 ^a^	0.079 ^a^	0.081 ^a^	0.095 ^b^	0.079 ^a^	0.079 ^a^	0.001	0.000
Pentadecanoic acid	C15:0	0.395 ^a^	0.382 ^a^	0.441 ^ab^	0.458 ^ab^	0.582 ^b^	0.552 ^b^	0.018	0.000
Pentadecenoic acid	C15:1	0.104 ^b^	0.036 ^a^	0.077 ^b^	0.072 ^ab^	0.096 ^b^	0.039 ^a^	0.005	<0.0001
Palmitic acid	C16:0	20.28 ^d^	20.23 ^cd^	19.49 ^abc^	19.27 ^ab^	19.88 ^bcd^	19.041 ^a^	0.105	<0.0001
Palmitoleic acid	C16:1	3.467 ^ab^	3.443 ^ab^	3.773 ^b^	3.578 ^ab^	3.162 ^a^	3.059 ^a^	0.062	0.003
Heptadecanoic acid	C17:0	0.161 ^a^	0.164 ^a^	0.179 ^b^	0.212 ^c^	0.225 ^d^	0.248 ^e^	0.006	<0.0001
Heptadecenoic acid	C17:1	0.104 ^ab^	0.091 ^ab^	0.088 ^a^	0.139 ^abc^	0.190 ^c^	0.149 ^bc^	0.008	<0.0001
Stearic acid	C18:0	7.645 ^bc^	7.444 ^ab^	7.652 ^bc^	7.222 ^a^	7.996 ^c^	8.046 ^c^	0.061	<0.0001
Oleic acid	C18:1n9	31.15 ^c^	29.39 ^b^	30.04 ^b^	29.45 ^b^	29.73 ^b^	27.43 ^a^	0.202	<0.0001
Linoleic acid	C18:2n6	28.92 ^a^	30.67 ^c^	29.97 ^b^	31.54 ^d^	28.89 ^a^	31.58 ^d^	0.195	<0.0001
Arachidic acid	C20:0	0.175 ^a^	0.217 ^b^	0.174 ^a^	0.207 ^ab^	0.196 ^ab^	0.225 ^b^	0.005	0.000
Linolenic acid	C18:3n6	0.000 ^a^	0.000 ^a^	0.000 ^a^	0.000 ^a^	0.002 ^a^	0.002 ^a^	0.000	0.558
α Linolenic acid	C18:3n3	0.478 ^a^	0.584 ^b^	0.572 ^b^	0.673 ^d^	0.633 ^c^	0.728 ^e^	0.014	<0.0001
Conjugated linoleic acid	C18:2	0.170 ^a^	0.105 ^a^	0.122 ^a^	0.160 ^a^	0.157 ^a^	0.107 ^a^	0.008	0.019
Octadecatetraenoic acid	C18:4n3	0.249 ^b^	0.195 ^a^	0.232 ^ab^	0.316 ^c^	0.411 ^d^	0.252 ^b^	0.012	<0.0001
Eicosadienoic acid	C20:2n6	0.196 ^d^	0.031 ^ab^	0.096 ^bc^	0.082 ^abc^	0.151 ^cd^	0.020 ^a^	0.012	<0.0001
Eicosatrienoic acid	C20:3n6	0.424 ^a^	0.492 ^b^	0.511 ^b^	0.486 ^b^	0.508 ^b^	0.625 ^c^	0.011	<0.0001
Erucic acid	C22:1n9	0.047 ^a^	0.062 ^ab^	0.077 ^bc^	0.075 ^bc^	0.094 ^c^	0.096 ^c^	0.004	<0.0001
Eicosatrienoic acid	C20:3n3	0.402 ^a^	0.470 ^bc^	0.458 ^b^	0.457 ^b^	0.509 ^c^	0.575 ^d^	0.010	<0.0001
Arachidonic acid	C20:4n6	2.553 ^a^	3.231 ^c^	3.076 ^bc^	2.727 ^ab^	2.967 ^bc^	3.966 ^d^	0.084	<0.0001
Docosadienoic acid	C22:(2n6)	0.150 ^c^	0.066 ^a^	0.119 ^b^	0.079 ^a^	0.128 ^bc^	0.066 ^a^	0.006	<0.0001
Docosatrienoic acid	C22:3n6	0.148 ^d^	0.049 ^ab^	0.101 ^c^	0.061 ^b^	0.105 ^c^	0.037 ^a^	0.007	<0.0001
Eicosapentaenoic acid	C20:5n3	0.236 ^e^	0.092 ^a^	0.146 ^c^	0.115 ^b^	0.199 ^d^	0.069 ^a^	0.010	<0.0001
Lignoceric acid	C24:0	0.344 ^d^	0.141 ^b^	0.275 ^c^	0.144 ^b^	0.249 ^c^	0.088 ^a^	0.015	<0.0001
Nervonic acid	C24:1n9	0.737 ^a^	0.942 ^c^	0.896 ^bc^	0.777 ^ab^	0.927 ^bc^	1.244 ^d^	0.031	<0.0001
Docosatetraenoic acid	C22:4n6	0.204 ^a^	0.277 ^bc^	0.225 ^ab^	0.230 ^ab^	0.278 ^bc^	0.324 ^c^	0.009	<0.0001
Docosapentaenoic acid	C22:5n3	0.126 ^a^	0.131 ^a^	0.133 ^a^	0.127 ^a^	0.145 ^a^	0.253 ^b^	0.008	<0.0001
Docosahexaenoic acid	C22:6n3	0.082 ^a^	0.093 ^a^	0.085 ^a^	0.083 ^a^	0.104 ^a^	0.131 ^b^	0.004	<0.0001
Other FAs		0.322 ^ab^	0.166 ^a^	0.211 ^a^	0.506 ^bc^	0.700 ^c^	0.296 ^ab^	0.040	<0.0001
Σ SFAs		29.66 ^bc^	29.30 ^bc^	28.91 ^ab^	28.17 ^a^	29.84 ^c^	28.88 ^ab^	0.117	<0.0001
Σ MUFAs		35.68 ^d^	34.04 ^b^	35.04 ^cd^	34.18 ^b^	34.27 ^bc^	32.09 ^a^	0.117	<0.0001
Σ PUFAs		34.34 ^a^	36.49 ^cd^	35.85 ^bc^	37.14 ^d^	35.19 ^ab^	38.73 ^e^	0.199	<0.0001
PUFAs/SFAs		1.159 ^a^	1.245 ^b^	1.240 ^b^	1.319 ^c^	1.179 ^a^	1.341 ^c^	0.252	<0.0001
Σ Ω3		1.572 ^a^	1.564 ^a^	1.626 ^a^	1.771 ^b^	2.001 ^c^	2.008 ^c^	0.032	<0.0001
Σ Ω6		32.59 ^a^	34.82 ^bc^	34.10 ^b^	35.21 ^c^	33.03 ^a^	36.61 ^d^	0.242	<0.0001
Ω6/Ω3		20.74 ^d^	22.28 ^e^	20.98 ^d^	19.88 ^c^	16.51 ^a^	18.25 ^b^	0.330	<0.0001
DFAs		77.66 ^a^	77.97 ^ab^	78.54 ^bc^	78.54 ^bc^	77.46 ^a^	78.87 ^c^	0.115	<0.0001
OFAs		20.73 ^cd^	20.76 ^d^	19.98 ^abc^	19.76 ^ab^	20.39 ^bcd^	19.53 ^a^	0.104	<0.0001
DFAs/OFAs		3.756 ^a^	3.757 ^a^	3.931 ^abc^	3.975 ^bc^	3.799 ^ab^	4.038 ^c^	0.025	0.0001

C:D, carbon number/double-bond number; SEM, standard error of the means; SFAs, saturated fatty acids; MUFAs, monounsaturated fatty acids; PUFAs, polyunsaturated fatty acids. DFAs—hypocholesterolemic fatty acids; OFAs—hypercholesterolemic fatty acids. Means in rows followed by the same letter are not significantly different at the 5% level of probability (*p* < 0.05).

**Table 5:** Lipid quality indices in thigh meat.

Vit E	*C. vulgaris*	DBI	IV	Cox	OS	PI	HPI	AI	TI	h/H
0	0	0.918 ^ab^	91.70 ^a^	3.393 ^a^	1385 ^a^	54.09 ^a^	3.165 ^a^	0.317 ^bc^	0.763 ^c^	3.171 ^a^
	1	0.929 ^abc^	94.40 ^b^	3.511 ^b^	1398 ^a^	58.02 ^b^	3.289 ^ab^	0.304 ^ab^	0.732 ^ab^	3.303 ^abc^
	2	0.961 ^d^	95.48 ^b^	3.689 ^c^	1521 ^c^	57.83 ^b^	3.347 ^b^	0.299 ^a^	0.703 ^a^	3.366 ^bc^
250	0	0.935 ^bc^	95.03 ^b^	3.579 ^b^	1472 ^b^	59.21 ^b^	3.143 ^a^	0.318 ^c^	0.748 ^bc^	3.173 ^a^
	1	0.914 ^a^	91.93 ^a^	3.410 ^a^	1392 ^a^	58.52 ^b^	3.157 ^a^	0.317 ^bc^	0.751 ^bc^	3.184 ^ab^
	2	0.940 ^c^	97.84 ^c^	3.684 ^c^	1525 ^c^	66.73 ^c^	3.354 ^b^	0.298 ^a^	0.714 ^a^	3.388 ^c^
Main effects										
Vit E										
0		0.936 ^a^	93.86 ^a^	3.531 ^a^	1435 ^a^	56.64 ^a^	3.267 ^a^	0.307 ^a^	0.733 ^a^	3.280 ^a^
250		0.929 ^a^	94.92 ^b^	3.557 ^a^	1463 ^b^	61.49 ^b^	3.218 ^a^	0.311 ^a^	0.738 ^a^	3.248 ^a^
*C. vulgaris*										
0		0.926 ^a^	93.36 ^a^	3.486 ^a^	1429 ^b^	56.65 ^a^	3.154 ^a^	0.317 ^b^	0.756 ^b^	3.172 ^a^
1		0.921 ^a^	93.15 ^a^	3.460 ^a^	1395 ^a^	58.27 ^ab^	3.223 ^a^	0.310 ^b^	0.742 ^b^	3.243 ^a^
2		0.951 ^b^	96.66 ^b^	3.686 ^b^	1523 ^c^	62.28 ^b^	3.351 ^b^	0.299 ^a^	0.709 ^a^	3.377 ^b^
*p*-Value										
Vit E		0.074	0.001	0.083	0.0005	<0.0001	0.100	0.102	0.350	0.367
*C. vulgaris*		<0.0001	<0.0001	<0.0001	<0.0001	<0.0001	<0.0001	<0.0001	<0.0001	0.0001
Vit E × *C. vulgaris*		0.0002	<0.0001	<0.0001	<0.0001	<0.0001	0.135	0.105	0.052	0.213
SEM										
Vit E		0.002	0.207	0.010	5.176	0.425	0.020	0.002	0.004	0.025
*C. vulgaris*		0.003	0.254	0.013	6.340	0.521	0.025	0.002	0.005	0.030
Vit E × *C. vulgaris*		0.004	0.359	0.018	8.966	0.737	0.035	0.003	0.007	0.042

DBI, double-bond index; IV, iodine value; Cox, calculated oxidizability value; OS, oxidative susceptibility; PI, peroxidisability index; HPI, health-promoting index; AI, atherogenic index; TI, thrombogenic index; h/H, the hypocholesterolemic/hypocholesterolemic ratio. Means in columns followed by the same letter are not significantly different at the 5% level of probability (*p* < 0.05).

**Table 6:** Effects of dietary treatments on the vitamin E concentration in the thigh muscle of broiler chickens.

Vit E	*C. vulgaris*	Vitamin E Concentration
0	0	55.927 ^a^
	1	73.913 ^bc^
	2	75.897 ^bc^
250	0	68.064 ^b^
	1	77.161 ^c^
	2	80.923 ^c^
Main effects		
Vit E		
0		68.579 ^a^
250		75.383 ^b^
*C. vulgaris*		
0		61.995 ^a^
1		75.537 ^b^
2		78.410 ^b^
*p*-Value		
Vit E		0.001
*C. vulgaris*		<0.0001
Vit E × *C. vulgaris*		0.094
SEM		
Vit E		1.168
*C. vulgaris*		1.431
Vit E × *C. vulgaris*		2.023

SEM, standard error of means. Means in columns followed by the same letter are not significantly different at the 5% level of probability (*p* < 0.05).

**Table 7:** Effects of dietary treatments on the markers of lipid oxidation of thigh muscle of broiler chickens.

		Primary Oxidation Products			Secondary Oxidation Products	
Vit E	*C. vulgaris*	CDs	CTs	PV	pA	TBARSs
0	0	22.88 ^b^	9.821 ^a^	0.691 ^b^	55.44 ^b^	367.7 ^c^
	1	16.87 ^ab^	6.120 ^a^	0.501 ^ab^	37.05 ^ab^	195.4 ^ab^
	2	16.62 ^a^	5.941 ^a^	0.482 ^a^	37.01 ^ab^	168.8 ^a^
250	0	20.09 ^b^	6.361 ^a^	0.541 ^b^	41.05 ^ab^	321.7 ^ab^
	1	15.46 ^ab^	5.682 ^a^	0.450 ^ab^	40.80 ^ab^	264.5 ^abc^
	2	15.29 ^a^	5.461 ^a^	0.431 ^a^	30.88 ^a^	202.1 ^ab^
Main effects						
Vit E						
0		18.79 ^a^	7.293 ^a^	0.552 ^a^	43.17 ^a^	243.9 ^a^
250		16.75 ^a^	5.833 ^a^	0.427 ^a^	37.58 ^a^	262.8 ^a^
*C. vulgaris*						
0		21.48 ^b^	8.089 ^a^	0.615 ^b^	48.25 ^b^	344.7 ^b^
1		16.17 ^ab^	5.904 ^a^	0.471 ^ab^	38.92 ^ab^	229.9 ^a^
2		15.96 ^a^	5.697 ^a^	0.457 ^a^	33.95 ^a^	185.4 ^a^
*p*-Value						
Vit E		0.301	0.230	0.101	0.118	0.467
*C. vulgaris*		0.028	0.211	0.026	0.010	0.0002
Vit E × *C. vulgaris*		0.929	0.497	0.896	0.122	0.193
SEM						
Vit E		1.224	0.830	0.027	2.406	17.90
*C. vulgaris*		1.498	1.017	0.033	2.947	21.92
Vit E × *C. vulgaris*		2.119	1.438	0.046	4.168	31.00

CDs, conjugated dienes; CTs, conjugated trienes; PV, peroxide value; pA, para-anisidine; TBARSs, thiobarbituric acid reactive substances; SEM, standard error of means. Means in columns followed by the same letter are not significantly different at the 5% level of probability (*p* < 0.05).

## Data Availability

The original contributions presented in the study are included in the article; further inquiries can be directed to the corresponding author.
